# CO_2_ availability influences hydraulic function of C_3_ and C_4_ grass leaves

**DOI:** 10.1093/jxb/ery095

**Published:** 2018-03-10

**Authors:** Samuel H Taylor, Michael J Aspinwall, Chris J Blackman, Brendan Choat, David T Tissue, Oula Ghannoum

**Affiliations:** 1 Hawkesbury Institute for the Environment, Western Sydney University, Penrith NSW, Australia; 2 Lancaster Environment Centre, University of Lancaster, Lancaster, UK; 3 Department of Biology, University of North Florida, Drive, Jacksonville, FL, USA; 4 ARC Centre of Excellence for Translational Photosynthesis, Australia

**Keywords:** C_4_ photosynthesis, glacial CO_2_, grasses, leaf gas exchange, leaf hydraulic conductance, osmotic adjustment, pressure–volume curve, stomatal conductance, turgor loss point

## Abstract

Atmospheric CO_2_ (*c*_a_) has increased since the last glacial period, increasing photosynthetic water use efficiency and improving plant productivity. Evolution of C_4_ photosynthesis at low *c*_a_ led to decreased stomatal conductance (*g*_s_), which provided an advantage over C_3_ plants that may be reduced by rising *c*_a_. Using controlled environments, we determined how increasing *c*_a_ affects C_4_ water use relative to C_3_ plants. Leaf gas exchange and mass per area (LMA) were measured for four C_3_ and four C_4_ annual, crop-related grasses at glacial (200 µmol mol^−1^), ambient (400 µmol mol^−1^), and super-ambient (640 µmol mol^−1^) *c*_a_. C_4_ plants had lower *g*_s_, which resulted in a water use efficiency advantage at all *c*_a_ and was broadly consistent with slower stomatal responses to shade, indicating less pressure on leaf water status. At glacial *c*_a_, net CO_2_ assimilation and LMA were lower for C_3_ than for C_4_ leaves, and C_3_ and C_4_ grasses decreased leaf hydraulic conductance (*K*_leaf_) similarly, but only C_4_ leaves decreased osmotic potential at turgor loss. Greater carbon availability in C_4_ leaves at glacial *c*_a_ generated a different hydraulic adjustment relative to C_3_ plants. At current and future *c*_a_, C_4_ grasses have advantages over C_3_ grasses due to lower *g*_s_, lower stomatal sensitivity, and higher absolute water use efficiency.

## Teaser

Carbon limitation and water use characteristics impact the relative performance of C_3_ and C_4_ grasses at past and future CO_2_

## Introduction

C_4_ photosynthetic pathways have evolved as solutions to photosynthetic inefficiencies linked with the oxygenation reaction of Rubisco (Sage, 2004; Sage *et al.*, 2012). Because of its potential for greater efficiency, engineered C_4_ photosynthesis has been proposed as a potential solution for improving global food security (von Caemmerer *et al.*, 2012), and C_4_ crops are leading contenders as sources of renewable biomass energy (Byrt *et al.*, 2011). Our understanding of C_4_ photosynthesis as an ecological adaptation is continuing to develop rapidly (Edwards and Smith, 2010; Lundgren *et al.*, 2015; Atkinson *et al.*, 2016; Watcharamongkol *et al.*, 2018). New insights into the timing and sequence of C_4_ evolution from phylogenetic studies have renewed debate about its expected physiological advantages (Edwards and Still, 2008; Edwards *et al.*, 2010; Christin *et al.*, 2011*b*, 2014; Sage *et al.*, 2011). Some C_4_ lineages probably arose during the Oligocene (~30 million years ago) but most arose over the last 20 million years, during the subsequent Miocene (Christin *et al.*, 2008, 2011*a*; Vicentini *et al.*, 2008; Besnard *et al.*, 2009; but see Kadereit *et al.*, 2012). During this period, ‘icehouse’ conditions of globally cooler temperatures and drier climates were linked with atmospheric CO_2_ concentrations (*c*_a_) lower than present day (Pagani *et al.*, 2005). Atmospheric CO_2_ has increased since the last glacial period, and consequent increases in photosynthetic water use efficiency have been associated with declines in water stress and improvements in plant productivity (Polley *et al.*, 1993; Mayeux *et al.*, 1997). It has been speculated that in addition to impacts on photosynthetic performance (Ehleringer *et al.*, 1997), hydraulic function in C_3_ and C_4_ plants was differentially affected at low atmospheric CO_2_ (Osborne and Sack, 2012) because of greater stomatal opening in C_3_ plants resulting in greater water stress (Polley *et al.*, 1993). It is also expected that under future, high CO_2_ climates, the combination of CO_2_ fertilization and improved water use efficiency will continue to influence the relative performance of C_3_ and C_4_ plants (Ghannoum *et al.*, 2000; Ainsworth and Long, 2005; Leakey, 2009). To establish whether C_4_ plants gain hydraulic advantages because of relatively small increases in stomatal conductance (*g*_s_) responding to *c*_a_ (Osborne and Sack, 2012), it is important to verify relative stomatal responses experimentally and investigate their impact on physiological function, including hydraulic properties.

Photosynthesis in C_4_ leaves is characterized by biochemical pumps that initially combine phosphoenolpyruvate (PEP) and CO_2_ to form C_4_ acids and subsequently transfer those acids, release CO_2_ in the presence of Rubisco, and recycle PEP (Edwards *et al.*, 2001; Sage, 2004). The initial biochemical step used to form C_4_ acids is highly efficient, and a high CO_2_ concentration at the site of Rubisco carboxylation minimizes photorespiration in C_4_ plants. Therefore, leaf internal CO_2_ concentrations (*c*_i_) are lower for CO_2_ compensation and photosynthetic saturation, and quantum yield can be greater (Pearcy and Ehleringer, 1984). Importantly for plant hydraulics, photosynthetic water use efficiency is consequently high (Pearcy and Ehleringer, 1984; Long, 1999). A central question has been whether this improved water use efficiency provides advantages for C_4_ plants over C_3_ plants in habitats with restricted water availability (Osmond *et al.*, 1982; Hattersley, 1983; Pearcy and Ehleringer, 1984). Recent comparative studies of the numerous C_4_ lineages in the grass family have supported the idea that their evolution and maintenance were often linked with improved performance in drier or more open habitats compared with C_3_ sister groups (Osborne and Freckleton, 2009; Edwards and Smith, 2010; Christin and Osborne, 2014). Osborne and Sack (2012) proposed that improved hydraulic safety, afforded by the evolution of lower *g*_s_ among C_4_ species (Osmond *et al.*, 1982; Taylor *et al.*, 2010, 2012), might have increased the potential of C_4_ grasses to colonize drier habitats when *c*_a_ was lower than it is today. They also noted that *g*_s_ is usually higher at glacial *c*_a_ compared with ambient *c*_a_, but the increase in *g*_s_ is less at glacial *c*_a_ in C_4_ plants than in C_3_ plants. Using steady-state models of coupled photosynthesis and plant hydraulics, they showed that lower *g*_s_ could have protected C_4_ plants from loss of hydraulic conductivity and allowed net CO_2_ assimilation (*A*) to be maintained as soil dried at low *c*_a_. They therefore proposed that in addition to biochemical advantages supporting higher *A* at low *c*_i_, protection of hydraulic function was an important advantage to C_4_ grasses at low *c*_a_.

Importantly, the models that Osborne and Sack (2012) used to predict hydraulic performance in C_3_ and C_4_ species at glacial *c*_a_ did not predict potential adjustments to co-ordination of leaf gas exchange and hydraulic function at low *c*_a_. Although evidence suggests that in non-woody species, decreased *g*_s_ at elevated *c*_a_ is associated with less negative leaf water potentials, lower hydraulic conductivity, and greater resistance to embolism, little is known about the influence of *c*_a_ on co-ordination between photosynthetic capacity and hydraulic function (Domec *et al.*, 2017). Changing irradiance results in parallel changes in leaf hydraulic conductance (*K*_leaf_) and photosynthetic capacity of woody C_3_ plants, optimizing leaf hydraulic function (Brodribb and Jordan, 2011; Carins Murphy *et al.*, 2012). In contrast, adjustment to high vapour pressure deficit (VPD) is linked with closure of stomata to protect hydraulic function (Carins Murphy *et al.*, 2014). In the case of *c*_a_, hydraulic demand is influenced by changes in *g*_s_ that compensate for carbon availability (Franks *et al.*, 2013). Because the economics of leaf structure–function relationships may depend on *c*_a_, it is likely that *c*_a_ has complex effects on co-ordination between *K*_leaf_ and *g*_s_. For instance, smaller, more densely packed stomata are sometimes observed at low *c*_a_ (Woodward, 1987; Woodward and Bazzaz, 1988; Franks and Beerling, 2009*b*), which may increase the sensitivity of *g*_s_ to VPD (Franks and Beerling, 2009*a*; Drake *et al.*, 2013), serving a protective function. Conversely, higher anatomical maxima for *g*_s_ observed at low *c*_a_ in sunflower, which were a result of larger, more densely packed stomata, were linked with greater xylem-specific conductivity, but the phloem ratio and hydraulic safety were decreased (Rico *et al.*, 2013). Photosynthetic type may further affect the impact of *c*_a_ on the relationships between hydraulic supply and demand because the carbon assimilation advantage provided by C_4_ photosynthesis may support additional flexibility in hydraulic adjustment. At ambient CO_2_, relative to C_3_ species, C_4_ dicots maintain *A* at relatively lower *g*_*s*_, and either increase hydraulic safety by decreasing xylem conduit diameter, or display greater leaf area for similar investments in stem xylem supply (Kocacinar and Sage, 2003, 2004; Kocacinar *et al.*, 2008).

In grasses, leaf hydraulic performance is particularly important: leaves contribute 50–72% of resistance along whole-plant hydraulic pathways (Meinzer *et al.*, 1992; Martre *et al.*, 2001). The relative sensitivities of *K*_leaf_ and *g*_s_ are also crucial in determining water use strategies among grasses. Both C_3_ and C_4_ grasses have been reported to show routine diurnal declines in leaf hydraulic conductivity when stomata do not close sufficiently to protect hydraulic function (Neufeld *et al.*, 1992; Holloway-Phillips and Brodribb, 2011*b*). Susceptibility to declines in conductivity is variable both among species and among cultivars (Holloway-Phillips and Brodribb, 2011*a*), and nocturnal root pressure and refilling of embolized vessels facilitates recovery from diurnal stress in some grass species (McCully, 1999; Holloway-Phillips and Brodribb, 2011*b*; Cao *et al.*, 2012; Gleason *et al.*, 2017). Protection against runaway declines in *K*_leaf_ can be provided by stomatal closure (Brodribb and Holbrook, 2003), and fast stomatal responses are considered a key characteristic of grasses (Hetherington and Woodward, 2003; Franks and Farquhar, 2007). Faster stomatal responses to light can improve intrinsic water use efficiency (iWUE=*A*/*g*_sw_, where *g*_sw_ is *g*_s_ for water) by producing a better match between rapid photosynthetic responses and the slower stomata, which may improve overall water use efficiency, resulting in greater conservation of soil water and thereby decreased hydraulic stress (Lawson and Blatt, 2014).

Our goal was to determine whether growth *c*_a_ had different impacts on leaf function in selected C_3_ and C_4_ annual grasses comparable with crop species. We predicted that to support increased transpiration at low *c*_a_, *K*_leaf_ would increase and turgor loss points would decrease to compensate for increased hydraulic demand. In addition, we determined whether rates of stomatal closure, responding to low light, increased at low *c*_a_. We anticipated that leaf mass per area (LMA) would decrease in plants with carbon limitation at low *c*_a_, and that decreases in iWUE and the extent of carbon limitation imposed by low *c*_a_ would be greater for C_3_ than for C_4_ species (Osborne and Sack, 2012). We therefore expected that leaf physiological responses to a range of *c*_a_ would be larger in C_3_ than in C_4_ grasses. Plants were grown in CO_2_ concentrations that represented: some of the lowest *c*_a_ conditions (~200 µmol mol^−1^) that occurred in the glacial period during which C_4_ grass lineages diversified (Edwards *et al.*, 2010); ambient *c*_a_ (400 µmol mol^−1^); and super-ambient *c*_a_ (640 µmol mol^−1^).

## Materials and methods

### Growth conditions

Plants were grown in walk-in climate-controlled growth chambers (Biochambers, Winnipeg, Manitoba) equipped with additive CO_2_, and CO_2_ scrubber equipment. Three *c*_a_ treatments were imposed: glacial (*c*_GLA_), 204 ± 27 μmol mol^−1^; ambient (*c*_AMB_), 408 ± 11 μmol mol^−1^; and super-ambient (*c*_SUP_) 640 ± 2 μmol mol^−1^ (mean ±SD; 72 daily means). The *c*_AMB_ and *c*_SUP_ treatments were rotated between cabinets 1 week prior to the first measurements, during the fourth week after sowing. The *c*_GLA_ treatment was maintained in a single cabinet throughout the experiment because of the technical demands of obtaining a stable CO_2_ concentration at glacial *c*_a_. Growing conditions were set to a night-time temperature of 18 °C and a daytime temperature of 26 °C, resulting in a daily mean temperature of 22 °C (mean ±SD for 72 daily means: *c*_GLA_, 21.9 ± 0.23; *c*_AMB_, 22.1 ± 0.21; and *c*_SUP_, 22.1 ± 0.28). Temperatures and light levels were ramped daily in two even steps between 06.00 h and 08.00 h and between 18.00 h and 20.00 h (14 h light:10 h dark). Light was supplied by HID lamps, which provided photosynthetic photon flux density (PPFD) at the top of the canopy that varied within each cabinet between 300 μmol m^−2^ s^−1^ and 650 μmol m^−2^ s^−1^; daily quantum inputs were ~21 mol m^−2^ d^−1^ (mean ±SD for 72 daily means: *c*_GLA_, 21.3 ± 1.8; *c*_AMB_, 21.5 ± 1.9; and *c*_SUP_, 21.5 ± 2.0). Mean daily values for relative humidity ranged from 60% to 83% (mean ±SD for 72 daily means: *c*_GLA_, 77 ± 8; *c*_AMB_, 78 ± 1; and *c*_SUP_, 76 ± 6), providing VPDs of ~0.74 kPa under daytime conditions, and ~0.46 kPa during the night.

### Plant material

Our study plants were eight annual grass species, four C_3_ and four C_4_, used as food crops or close relatives of species used as food crops. In Poacaeae, all C_4_ grasses belong to a clade referred to as PACMAD (Aliscioni *et al.*, 2012); within PACMAD two C_4_ crop species have been domesticated from wild relatives in the Chloridoideae (teff and finger millet), and several from the Panicoideae subfamily (Christin *et al.*, 2009). Grasses with C_3_ photosynthesis used as grain crops originate in the subfamilies Pooideae and Oryzoideae, which belong to a separate clade currently referred to as BEP (Kellogg, 1998; Aliscioni *et al.*, 2012). Relevant Chloridoideae species could not be obtained, so we only used C_4_ grasses from the Panicoideae. *Sorghum bicolor* (great millet), *Setaria italica* (foxtail millet), and *Digitaria exilis* (fonio millet) represent independent evolutionary origins of the NADP-malic enzyme (NADP-ME) subtype of C_4_ photosynthesis (Aliscioni *et al.*, 2012); *Panicum miliaceum* (proso millet) represents the NAD-ME C_4_ subtype (Giussani *et al.*, 2001; Aliscioni *et al.*, 2012). C_3_ species were *Panicum bisulcatum* and *Steinchisma laxa* (two wild relatives from Panicoideae), *Triticum turgidum* (durum wheat, Pooideae), and *Oryza sativa* ssp. *japonica* (rice, Ehrhartoideae; Table 1).

**Table 1.** Sources and phylogenetic placement of study species

Species	Photosynthetic type^*a*^	Phylogenetic placement^*a*^	Accession (source)
*Triticum turgidum* L. ssp. *durum*	C_3_	Pooideae	AUS-26564 /PERSIA128 (Tony Condon, CSIRO Agriculture, ACT)
*Oryza sativa* L. ssp. *japonica* Kato	C_3_	Ehrhartoideae	IAC1131 (Brian Atwell, Macquarie University, Sydney NSW)
*Panicum bisulcatum* Thunb.	C_3_	Panicoideae: Paniceae	(Ghannoum laboratory)
*Steinchisma laxa* (Sw.) Zuloaga	C_3_	Panicoideae: Paspaleae	(Ghannoum laboratory)
*Sorghum bicolor* (L.) Moench	C_4_	Panicoideae: Andropogoneae	Tx623 (Alan Cruickshank, Department of Agriculture and Fisheries, Hermitage Research Facility, Warwick QLD)
*Setaria italica* (L.) P.Beauv.	C_4_	Panicoideae: Paniceae: Cenchrinae	AusTRCF 108040 (AusPGRIS: Tropical Crops and Forages Collection)
*Digitaria exilis* (Kippist) Stapf	C_4_	Panicoideae: Paniceae: Anthephorinae	AusTRCF 108024/PDE7 (AusPGRIS: Tropical Crops and Forages Collection)
*Panicum miliaceum* L.	C_4_	Panicoideae: Paniceae: Panicinae	(Ghannoum laboratory)

^*a*^ Aliscioni *et al.* (2012).

Plants were grown from seed in Osmocote Professional Seed Raising & Cutting Mix (Scotts Australia Pty Ltd, Bella Vista, NSW) in 0.55 litre plastic square tubes (Garden City Plastics, Somersby NSW: top dimension 70 × 70 mm, 160 mm deep). Seeds were sown directly into six pots and germinated under the different CO_2_ treatments; the number of plants per pot and the size of plants varied depending on the species. To allow for balanced sampling and to account for within-cabinet variability, at germination, pots were arranged into a fully randomized blocked design with one pot from every species in each block. The pots were checked daily and watered as necessary to prevent surface drying. To minimize root binding, roots were allowed to grow out of pots into a layer of wetted Scoria. To minimize nutrient limitation, plants were fed with a complete fertilizer (Thrive All Purpose Soluble Plant Food, Yates, Auckland, New Zealand) every 2–3 weeks during the course of the experiment.

### Steady-state gas exchange and stomatal response to PPFD

We measured gas exchange using six LI-6400XT photosynthesis systems (LI-COR Inc., Lincoln NE, USA) equipped with CO_2_ mixers (LI-6400-01) and 2 × 3 cm red-blue LED light sources (LI-6400-02B). Pairs of LI-6400XT machines were randomly allocated to the three *c*_a_ treatments and were rotated every 2 d: each pair of machines was used to measure two of the six blocks in every cabinet over the course of the experiment. Measurements were made under the growth conditions. To minimize disruption of *c*_a_ treatments, the cuvette and integrated gas analysers of the LI-6400XT were placed inside the growth chambers and consoles outside the growth chambers (growth chambers were opened briefly before and after switching leaves). Measurements were conducted on the mid-section of individual, recently expanded leaves inserted parallel to the long axis of the 2 × 3 cm chamber, and leaf areas were calculated as cuvette length×average leaf width, measured to the nearest 0.5 mm with a ruler. Leaves were allowed to come to steady state [showing no systematic trends with a coefficient of variation (CV) <0.1 over a 5 min period] at a PPFD of 500 μmol m^−2^ s^−1^ (growth light levels) and cuvette CO_2_ concentrations matched to *c*_a_ at the time of measurement: (*c*_GLA_, 184 ± 4 μmol mol^−1^; *c*_AMB_, 406 ± 5 μmol mol^−1^; and *c*_SUP_, 647 ± 6 μmol mol^−1^; mean ±SD ≥43 leaves, CV for individual leaves <5%). Relative humidity was maintained at ~70% and block temperature at 26 °C, resulting in leaf VPDs of 1 ± 0.07 kPa (mean ±SD, *n*=137 leaves; CV for individual leaves <8.1%). An auto-program (logging every 10 s) was used to record initial steady-state values for gas exchange (*A*, *g*_sw_, and iWUE), followed by the response of *g*_*sw*_ to a step-change decrease in light availability from 500 μmol m^−2^ s^−1^ to 100 μmol m^−2^ s^−1^ PPFD. The rate of stomatal response to PPFD (Δ*g*_sw_/Δ*t*) was characterized based on the magnitude (Δ*g*_sw_) and duration (Δ*t*) of the initial decrease in *g*_sw_.

### Leaf hydraulic conductance and LMA

Because C_3_ and C_4_ species differed in the response of *A* to *c*_a_ between *c*_GLA_ and *c*_AMB_, we determined *K*_leaf_ in those treatments using the evaporative flux method (Sack *et al.*, 2002). Cut stems were transported to the lab in water, where flag or second-leaf laminas were excised and, using parafilm, were sealed onto parafilm-wrapped, cylindrical plastic rods. The rod and leaf were submerged, and the leaf re-cut and positioned in water-filled Tygon tubing linked to a reservoir of de-gassed Milli-Q water on a balance (CPA225D, Sartorius, Göttingen, Germany; 10 µg accuracy). The seal was tested by pressure from a 100 ml syringe that was used to fill and empty the system and was attached to the Tygon tubing using Luer fittings. Leaves were supported using fishing line stretched across a wooden frame, with their adaxial surface uppermost and parallel with the meniscus in the reservoir. Transpiration was induced using a desk fan and a lamp (leaf surface PPFD 100–150 μmol m^−2^ s^−1^). Every second, output from the balance was logged to a computer and plotted to determine when transpiration (*E*, mol m^−2^ s^−1^) obtained a steady state for 5 min. At steady state, the temperature of the abaxial surface of the leaf (T_leaf_) and air (T_air_, shaded) were established using two type-K thermocouples and a Pico TC-08, then the leaf was immediately sheathed in plastic and cut at its base. After 15 min equilibration, a Scholander pressure bomb (PMS 1505D with grass compression gland; PMS Instrument Company, Albany, OR, USA) was used to determine water potential, which estimated the leaf hydrostatic gradient (ΔΨ). Leaf area for these leaves (and an extra set collected from the *c*_SUP_ treatment) was determined using a Canon LiDE 510 flatbed scanner and ImageJ software (Abràmoff *et al.*, 2004). We calculated *K*_leaf_ as *E*/(area×ΔΨ).

LMAs (g m^−2^) were calculated using dry masses determined after a minimum of 48 h drying at 65 °C.

### Pressure–volume relationships

Pressure–volume (P–V) relationships were determined using bench-drying. On the morning of measurement, attached flag leaves were sealed into plastic bags containing exhaled breath and were allowed to equilibrate for a minimum of 40 min to quench transpiration and ensure high turgor. Leaves sheathed in this manner were subsequently excised at the base of the lamina and moved to the laboratory. Initially, leaves remained sealed in plastic between measurements of fresh mass (FM, g) and water potential (Ψ; Scholander pressure bomb). As water potential declined, leaves were occasionally removed from the plastic for short periods to increase the rate of drying. A minimum of 20 min equilibration was ensured between pressure bomb measurements. At the conclusion of FM and Ψ measurements, leaves were dried for a minimum of 48 h at 65 °C to determine dry mass (DM, g). The turgid mass (TM, g) was estimated by extrapolation of the initial linear FM–Ψ relationship (Kubiske and Abrams, 1990) and used to calculate relative water contents (RWC_S_) for entire leaves as (FM−DM)/(TM−DM), and leaf dry matter content (LDMC=DM/TM).

We optimized parameter selection for P–V relationships of individual leaves by minimizing the absolute difference between estimates of osmotic potential at full turgor (π_0_, MPa) obtained below (π_0,1_) and above (π_0,2_) turgor loss, comparing all possible combinations that could be fit for each leaf within our data set. First, below-turgor loss fits for 1/Ψ=*a*(1−RWC)+π_0,1_ (linear regression with slope *a*, and *y*-intercept π_0,1_) were obtained from all sequences representing at least three of the smallest RWC values and excluding two or more of the highest RWC values. Next, the *x*-intercept of the below-turgor loss relationship (apoplastic fraction, a_f_) was used to establish the RWC of the symplasm [RWC_S_=(RWC−a_f_)/(1−a_f_)]. Then the osmotic potential, π=1/[*a*(1−RWC_S_)+π_0,1_] (MPa) and turgor pressure, Ψ_P_=Ψ−π (MPa) were derived for the complementary above-turgor loss data. Finally, the bulk modulus of elasticity (ε, MPa) and π_0,2_ were obtained from linear regression of Ψ_P_= −ε(1–RWC_S_)−π_0,2_. Turgor loss point characteristics were calculated for the pair of linear relationships where [π_0,1_−π_0,2_] was smallest, and 0<a_f_<1. Using π_0,1_ to estimate π_0_, the RWC at turgor loss (RWC_TLP_) was established by determining the RWC_S_ at which Ψ_P_=0, and was used to predict osmotic potential at turgor loss (π_TLP_, MPa) from the equation for π.

### Statistical analysis

Statistics were calculated using R Language and Environment (R Core Team, 2016; https://www.R-project.org/). We fit linear models (*lm*) of species×*c*_a_ responses, which we minimized using the Akaike information criterion (AIC). Where species effects were significant (or marginally so), we used linear contrasts to compare species means by photosynthetic type. Tests of photosynthetic type effects are approximate because the C_3_/C_4_ comparisons were not phylogenetically independent, and only a small number of species were included in our experiment. To adjust for heteroskedasticity, log_e_ transformation was applied to values for *g*__sw__, iWUE, and *K*_leaf_. Heteroskedasticity in Δ*g*_sw_/Δ*t* and P–V parameters could not be eliminated using transformation. For these parameters, we applied non-parametric Kruskal–Wallis tests, and present median values.

## Results

### Impact of *c*_a_ on steady-state leaf gas exchange

iWUE was more responsive to the difference between *c*_GLA_ and *c*_AMB_ than the difference between *c*_AMB_ and *c*_SUP_ (Fig. 3A; especially on a relative scale, Table 2). At *c*_GLA_, iWUE was 33–74% lower (mean 56%) than at *c*_AMB_, while iWUE at *c*_SUP_ was 4–56% higher (mean 30%) than at *c*_AMB_. Differences in iWUE were significant for comparisons between *c*_GLA_ and *c*_SUP_ in every species (Tukey’s HSD, *P*≤0.0016), and between *c*_GLA_ and *c*_AMB_ in every species except *T. turgidum* (Tukey’s HSD, *P*≤0.0001; *T. turgidum*, *P*=0.11); differences between *c*_AMB_ and *c*_SUP_ were not significant (*P*≥0.077).

**Table 2.** Impact of photosynthetic type on leaf gas exchange relative to ambient CO_2_ (*c*_AMB_ ~400 μmol mol^−1^), at glacial CO_2_ (*c*_GLA_ ~200 μmol mol^−1^), and at super-ambient CO_2_ (**c**_SUP_ ~640 μmol mol^−1^)

Photosynthetic type (C_4_ subtype)	Species	iWUE (µmol mol^−1^)		Net CO_2_ assimilation (µmol m^−2^ s^−1^)		Stomatal conductance (mol m^−2^ s^−1^)	
		*c*_GLA_:*c*_AMB_	*c*_SUP_:*c*_AMB_	*c*_GLA_:*c*_AMB_	*c*_SUP_:*c*_AMB_	*c*_GLA_:*c*_AMB_	*c*_SUP_:*c*_AMB_
C_3_	*T. turgidum*	0.66	1.35	0.51	1.07	0.77	0.79
C_3_	*O. sativa*	0.37	1.3	0.63	1.2	1.72	0.93
C_3_	*S. laxa*	0.36	1.32	0.64	1.16	1.78	0.88
C_3_	*P. bisulcatum*	0.26	1.28	0.58	0.81	2.21	0.63
C_4_ (NADP-ME)	*S. bicolor*	0.4	1.39	0.95	1.05	2.34	0.76
C_4_ (NADP-ME)	*D. exilis*	0.53	1.04	0.87	0.92	1.65	0.88
C_4_ (NADP-ME)	*S. italica*	0.42	1.13	0.93	0.94	2.23	0.83
C_4_ (NAD-ME)	*P. miliaceum*	0.51	1.56	0.93	1.11	1.83	0.71
Kruskal–Wallis *P* C_3_/C_4_ (df=1)		NS	NS	*	NS	NS	NS

*P<0.05.

**Fig. 1.**
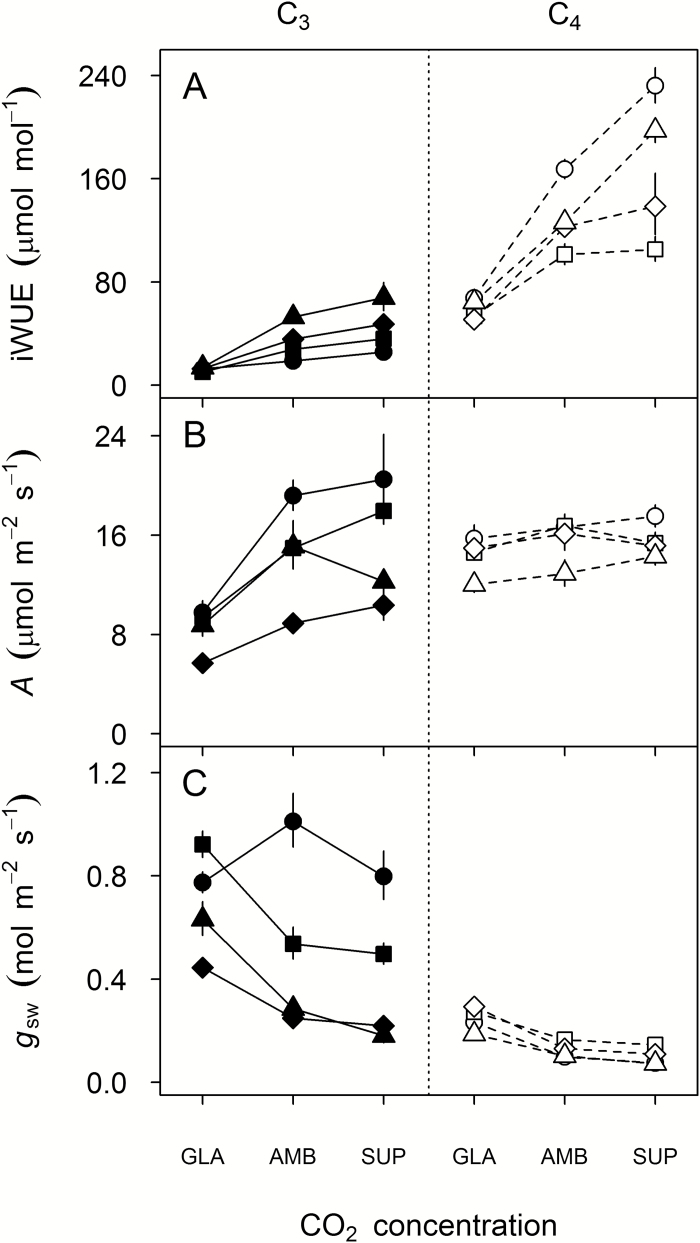
Response of steady-state leaf gas exchange to growth *c*_a_ (GLA, ~200 μmol mol^−1^; AMB, ~400 μmol mol^−1^; SUP, ~640 μmol mol^−1^), measured at growth PPFD (500 μmol m^−2^ s^−1^) and moderate vapour pressure deficit. (A) Intrinsic water use efficiency (iWUE); (B) net CO_2_ assimilation rate (*A*); and (C) stomatal conductance to water (*g*_sw_). Values are the mean ±SE (*n*=5–6) for eight crop and crop-related annual grass species, plotted by photosynthetic type: C_3_ (filled: circles, *Triticum turgidum*; squares, *Oryza sativa*; diamonds, *Steinchisma laxa*; triangles, *Panicum bisulcatum*) or C_4_ (open: circles, *Sorghum bicolor*; squares, *Digitaria exilis*; diamonds, *Setaria italica*; triangles, *Panicum miliaceum*).

C_4_ species showed much larger absolute decreases in iWUE than C_3_ species (Fig. 1A), linked with a marginally significant contrasts term for photosynthetic type×*c*_a_ (*t*_113_, *P*=0.048). At every *c*_a_, iWUE was always higher in C_4_ species (range 51–232 μmol mol^−1^) compared with C_3_ species (13–68 μmol mol^−1^; Fig. 1A). Relative changes in iWUE were not significantly different between C_3_ and C_4_ grasses, but this comparison was strongly influenced by C_3_*T. turgidum* (Table 2)*. Triticum turgidum* showed only a small reduction in iWUE from *c*_AMB_ to *c*_GLA_ and, surprisingly, decreased *g*_sw_ at *c*_GLA_ compared with *c*_AMB_ (Fig. 1B). The remaining three C_3_ grasses showed larger relative decreases in iWUE (63–74%) than any of the four C_4_ grasses (47–60%; Table 2).

Under the light conditions provided by our controlled-environment cabinets, which were non-saturating for photosynthesis (maximum 650 μmol m^−2^ s^−1^ PPFD), *A* was not higher in C_4_ species compared with C_3_ species at *c*_AMB_ and *c*_SUP_ (Fig. 1B), so the higher iWUE of C_4_ species at those CO_2_ concentrations was primarily due to lower *g*_sw_ (Fig. 1C). Higher iWUE among C_4_ grasses at *c*_GLA_ was primarily due to smaller relative reductions in *A* between *c*_AMB_ and *c*_GLA_ for C_4_ grasses (5–13%) compared with C_3_ grasses (36–49%); over the same range, relative increases in *g*_sw_ were comparable for C_3_ and C_4_ species (C_3_ excluding *T. turgidum*, +72–121%; *T. turgidum* −23%; C_4_, +65–134%; Table 2).

### Impact of *c*_a_ on dynamic leaf gas exchange

**Fig. 2.**
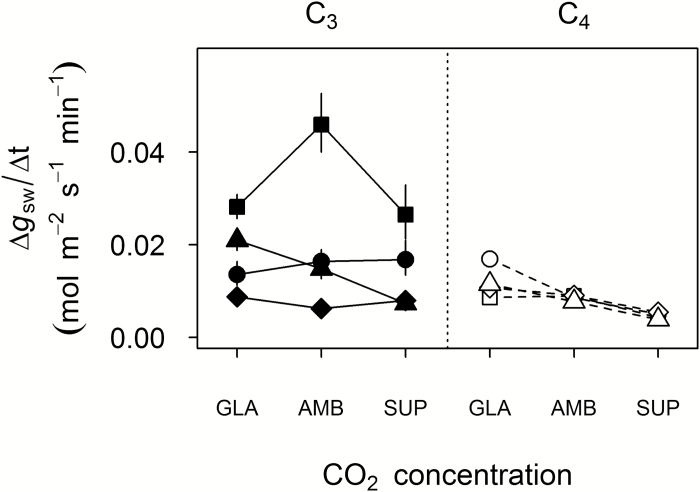
Effect of growth *c*_a_ (GLA, ~200 μmol mol^−1^; AMB, ~400 μmol mol^−1^; SUP, ~640 μmol mol^−1^) on rate of stomatal response to shade: a step-change decrease in PPFD from 500 μmol m^−2^ s^−1^ to 100 μmol m^−2^ s^−1^ (Δ*g*_sw_/Δ*t*) at a steady vapour pressure deficit. Values are the mean ±SE (*n*=5–6) for eight crop and crop-related annual grass species, plotted by photosynthetic type: C_3_ (filled: circles, *Triticum turgidum*; squares, *Oryza sativa*; diamonds, *Steinchisma laxa*; triangles, *Panicum bisulcatum*) or C_4_ (open: circles, *Sorghum bicolor*; squares, *Digitaria exilis*; diamonds, *Setaria italica*; triangles, *Panicum miliaceum*).

The rate of decrease in *g*_sw_ responding to a step decrease in PPFD from 500 μmol m^−2^ s^−1^ to 100 μmol m^−2^ s^−1^ (Δ*g*_sw_/Δ*t*) was generally greater for C_3_ than C_4_ grasses (Fig. 2). This was broadly consistent with the higher steady-state *g*_sw_ of C_3_ species at a PPFD of 500 μmol m^−2^ s^−1^ (Fig. 1C). A significant *c*_a_×species interaction was detected (*F*_14,113_, *P*<0.0001), associated with significant photosynthetic-type effects on the average response between *c*_GLA_ and *c*_AMB_ (*t*_113_, *P*=0.041), and between *c*_GLA_ and *c*_SUP_ (*t*_113_, *P*<0.0001). All C_4_ species increased Δ*g*_sw_/Δ*t* with decreasing *c*_a_, but only one C_3_ species (*P. bisulcatum*) showed a similar trend (Fig. 2). Pairwise tests for responses of Δ*g*_sw_/Δ*t* within species were significant only when comparing *c*_GLA_ and *c*_SUP_ of three species: *P. bisulcatum* (C_3_, *P*<0.001), *S. bicolor* (C_4_ NADP-ME, *P*<0.001), and *P. miliaceum* (C_4_ NAD-ME, *P*=0.004). Within their respective photosynthetic types, these three species showed the greatest values for iWUE and lowest values for *g*_sw_ at *c*_SUP_, and the greatest decreases in iWUE from *c*_SUP_ to *c*_GLA_ (Fig. 1A, C).

### Impact of *c*_a_ on LMA

**Fig. 3.**
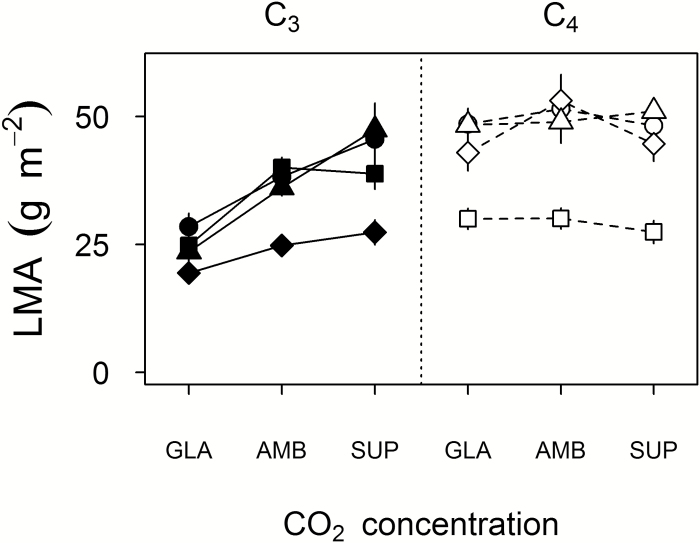
Response of leaf mass per area (LMA, individual upper canopy leaf lamina) to growth *c*_a_ (GLA, ~200 μmol mol^−1^; AMB, ~400 μmol mol^−1^; SUP, ~640 μmol mol^−1^). Values are the mean ±SE (*n*=5–6) for eight crop and crop-related annual grass species, plotted by photosynthetic type: C_3_ (filled: circles, *Triticum turgidum*; squares, *Oryza sativa*; diamonds, *Steinchisma laxa*; triangles, *Panicum bisulcatum*) or C_4_ (open: circles, *Sorghum bicolor*; squares, *Digitaria exilis*; diamonds, *Setaria italica*; triangles, *Panicum miliaceum*).

**Fig. 4.**
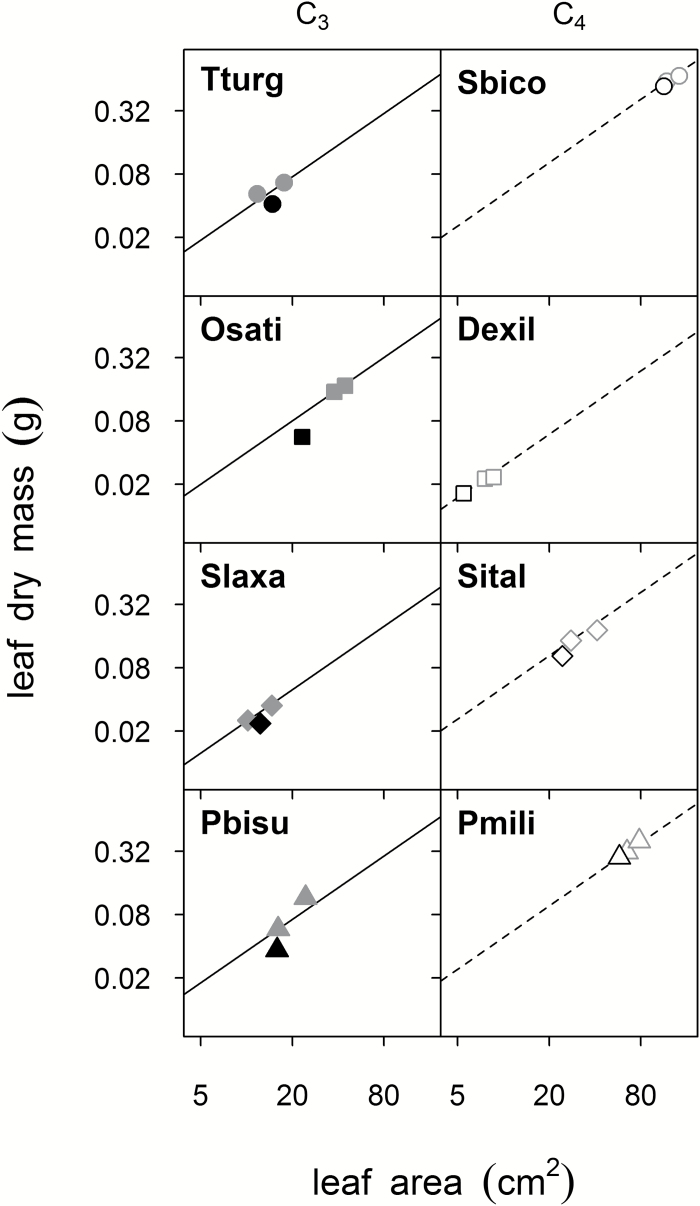
Effect of *c*_GLA_ (black symbols, versus grey for *c*_AMB_ and *c*_SUP_) on proportionality between leaf mass and area for individual upper canopy leaves (log–log allocation plots). Crop and crop-related annual grass species are plotted by photosynthetic type: C_3_ (filled: circles, *Triticum turgidum*; squares, *Oryza sativa*; diamonds, *Steinchisma laxa*; triangles, *Panicum bisulcatum*) or C_4_ (open: circles, *Sorghum bicolor*; squares, *Digitaria exilis*; diamonds, *Setaria italica*; triangles, *Panicum miliaceum*). Points are means (*n*=4–6); SE is omitted for clarity. Lines have slope=1, and the intersect is the mean value at *c*_AMB_.

At *c*_SUP_, LMA values for flag leaves of C_3_ and C_4_ species were similar (Fig. 3); however, the response of LMA to *c*_a_ differed among the eight species (Fig. 3; *F*_14,102_, *P*=0.0004). None of the C_4_ species exhibited significant changes in LMA in response to *c*_a_ (Tukey’s HSD, *P*>0.72). In the C_3_ species, LMA was similar across the three *c*_a_ treatments for *S. laxa*, but *T. turgidum*, *P. bisulcatum*, and *O. sativa* all showed significant reductions in LMA from either *c*_SUP_ to *c*_GLA_ (*T. turgidum* and *P. bisulcatum*, Tukey’s HSD *P*≤0.034) or from *c*_AMB_ to *c*_GLA_ (*O. sativa*, *P*=0.039; Fig. 3). The contrasts term for photosynthetic type×*c*_a_, which was statistically significant (*t*-test_102_, *P*=0.014), was therefore broadly associated with less sensitivity of LMA to *c*_a_ among the C_4_ species. Conservation of LMA across CO_2_ treatments in most C_4_ species was linked with proportionate decreases in mass and area of the flag leaves as *c*_a_ was reduced. Among C_3_ species, decreases in LMA arose because flag leaf mass decreased with *c*_a_ from *c*_SUP_ to *c*_GLA_, and flag leaf area decreased from *c*_SUP_ to *c*_AMB_, but leaf areas were often similar at *c*_GLA_ and *c*_AMB_ (Fig. 4).

### Response of *K*_leaf_ and P–V characteristics to decreases in *c*_a_ from *c*_AMB_ to *c*_GLA_

**Fig. 5.**
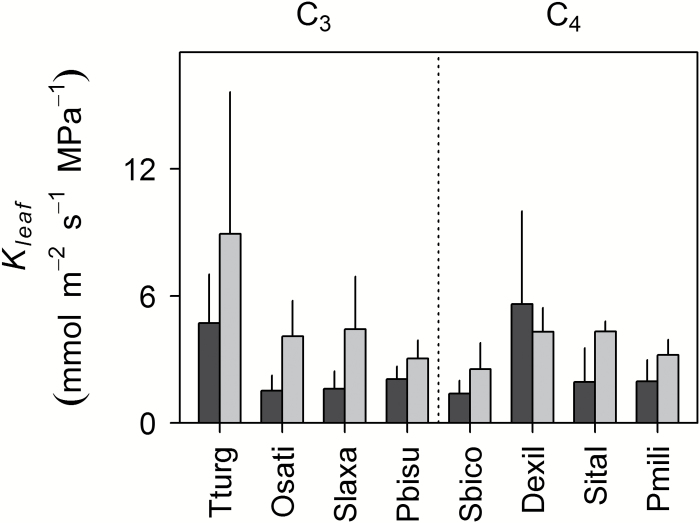
Response of leaf hydraulic conductance (*K*_leaf_) to growth at glacial (*c*_GLA_, ~200 μmol mol^−1^: dark shading) versus ambient (*c*_AMB_, ~400 μmol mol^−1^: light shading) CO_2_. Values are the mean ±SE (*n*=5) for eight crop and crop-related annual grass species differing in photosynthetic type. C_3_: Tturg, *Triticum turgidum*; Osati, *Oryza sativa*; Slaxa, *Steinchisma laxa*; Pbisu, *Panicum bisulcatum*. C_4_: Sbico, *Sorghum bicolor*; Dexil, *Digitaria exilis*; Sital, *Setaria italica*; Pmili, *Panicum miliaceum*.

There were no significant species×*c*_a_ effects on *K*_leaf_ (species×*c*_a_*F*_7,64_, *P*=0.814); however, on average, *K*_leaf_ was lower in plants grown at *c*_GLA_ (*F*_1,64_, *P*=0.005). The exception was *D. exilis*, a C_4_ species with small leaves, for which measurement errors were large (Fig. 5A).

In the P–V analysis, the response of LDMC to *c*_a_ was consistent with that of LMA measured during determination of *K*_leaf_. LDMC was not significantly different between the photosynthetic types at either *c*_a_, but was lower at *c*_GLA_ among C_3_ and not C_4_ leaves (Table 3). LDMC decreased by 4–11% in C_3_ grasses grown at *c*_GLA_, but C_4_ species showed no adjustment to *c*_GLA_ or increased LDMC by ≤3% at *c*_GLA_. This difference in average LDMC responses to *c*_a_ was statistically significant when comparing C_3_ and C_4_ species (Table 3). Despite these differences in LDMC responses between C_3_ and C_4_ species, we found no evidence for significant effects of photosynthetic type on the response of ε or RWC_TLP_ to *c*_a_. In contrast, the median π_TLP_ differed between C_3_ and C_4_ grasses at *c*_AMB_ but not at *c*_GLA_, linked with a significant effect of photosynthetic type (Table 3). At *c*_AMB_, π_TLP_ was less negative among C_4_ species (C_4_, −0.72 MPa to −0.87 MPa; C_3_, −0.94 MPa to −1.36 MPa). This difference was eliminated at *c*_GLA_ because only C_4_ grasses decreased π_TLP_ to more negative values (C_4_, −0.79 MPa to −1.27 MPa; C_3_, −0.91 MPa to −1.21 MPa; Table 2).

**Table 3.** Impact of growth *c*_a_ on pressure–volume curve characteristics and leaf dry matter content (medians, *n*=3–5; *c*_GLA_, glacial CO_2_ ~200 μmol mol^−1^; *c*_AMB_, ambient CO_2_ ~400 μmol mol^−1^)

Photosynthetic type (C_4_ subtype)	Species	Modulus of elasticity (ε, MPa)			Osmotic potential at turgor loss (π_TLP_, MPa)			RWC at turgor loss (RWC_TLP_, %)			Leaf dry matter content (LDMC, %)		
		*c*_GLA_	*c*_AMB_	Difference in median: *c*_GLA_−*c*_AMB_	*c*_GLA_	*c*_AMB_	Difference in median: *c*_GLA_−*c*_AMB_	*c*_GLA_	*c*_AMB_	Difference in median: *c*_GLA_−*c*_AMB_	*c*_GLA_	*c*_AMB_	Difference in median: *c*_GLA_−*c*_AMB_
C_3_	*T. turgidum*	11	9.4	1.6	−1.17	−1.31	0.14	94.8	94.5	~0	17	26	−8*
C_3_	*O. sativa*	5.4	5.7	−0.3	−1.21	−1.36	0.15	94.2	89.4	4.8	24	28	−4
C_3_	*S. laxa*	8	7.1	0.9	−1.13	−1.01	−0.12	93.3	92.3	1	18	29	−11*
C_3_	*P. bisulcatum*	4.1	6.2	−2.1	−0.91	−0.94	0.04	89.4	93.9	−4.5	26	32	−6^†^
C_4_ (NADP-ME)	*S. bicolor*	7.7	5.1	2.6	−1.03	−0.82	−0.2	97	97	0	26	23	3
C_4_ (NADP-ME)	*D. exilis*	5.9	9.4	−3.5	−0.79	−0.72	−0.08	92.1	93.6	−1.5	19	19	0
C_4_ (NADP-ME)	*S. italica*	8.1	7.4	0.7	−1.27	−0.87	−0.41**	93.2	95.7	−2.5	28	26	2
C_4_ (NAD-ME)	*P. miliaceum*	6.5	7.3	−0.8	−1	−0.85	−0.18	94.8	95.8	−1	31	29	2
Kruskal–Wallis *P* species (df=7)		NS	NS	–	**	***	–	NS	*	–	**	**	–
Kruskal-Wallis *P* C_3_/C_4_ (df=1)		NS	NS	NS	NS	*	*	NS	NS	NS	NS	NS	*

*P<0.05; **0.01<*P*<0.05; ***0.001<*P*<0.01; ^†^*P*<0.05 after exclusion of an extreme value >40% from *c*_GLA_ treatment that led to a median of 22% and difference in median of 10%

## Discussion

We exposed C_3_ and C_4_ grasses to atmospheric CO_2_ concentrations ranging from levels that occurred during the last 30 million years, when C_4_ lineages evolved and diversified, to those that could be experienced in the coming centuries. Across the range of *c*_a_, we expected that C_4_ species would maintain an iWUE advantage and show smaller physiological adjustments. Our results broadly support this expectation: the absolute response of *g*_sw_ to *c*_a_ was greater among C_3_ than among C_4_ grasses; and, as *c*_a_ decreased from *c*_AMB_ to *c*_GLA_, *A*, LDMC, and LMA declined more among C_3_ than among C_4_ species. Investigation of leaf hydraulic function at *c*_AMB_ and *c*_GLA_ showed that at *c*_GLA_, *K*_leaf_ decreased in both C_3_ and C_4_ species; and π_TLP_ of C_4_ leaves became more negative, hence more similar to π_TLP_ of C_3_ leaves, which did not adjust. Assaying the stomatal response to shade showed that higher steady-state *g*_sw_ of C_3_ species was linked with more rapid adjustment of *g*_sw_ to match *A*. Rates of stomatal closure were slightly more similar for C_3_ and C_4_ species at low *c*_a_, driven by strong responses of species that achieved high iWUE at elevated *c*_a_. These new findings are consistent with the hypothesis that carbon limitation is an important factor influencing leaf hydraulic function at different atmospheric [CO_2_]. Although there was substantial variation among species, photosynthetic type affected how leaf dry matter was deployed and how leaf turgor characteristics responded to *c*_GLA_.

### Gas exchange responses to *c*_a_

Steady-state gas exchange measurements provided the expected outcomes: *g*_s_ usually increased as *c*_a_ decreased (Osborne and Sack, 2012; Franks *et al.*, 2013); high *g*_s_ of C_3_ grasses was associated with greater *g*_s_ responses to *c*_a_; and low *g*_sw_ of C_4_ leaves resulted in higher iWUE at all levels of *c*_a_. Importantly, *A* declined for C_3_ but not C_4_ grasses at *c*_GLA_. Greater *A* among some C_3_ species compared with C_4_ species at *c*_AMB_ and *c*_SUP_ suggested that C_4_ photosynthetic performance may have been limited by PPFD, so the iWUE advantage to C_4_ species may underestimate advantages to C_4_ species that could arise at higher irradiances (Osmond *et al.*, 1982).

C_3_ grass leaves generally closed their stomata more quickly than C_4_ leaves in response to shade. The higher steady-state *g*_sw_ of C_3_ leaves may partially explain this difference between the photosynthetic types, but closer inspection of the data shows that Δ*g*_sw_/Δ*t* did not parallel the steady-state *g*_sw_ for species within each photosynthetic type. Interestingly, among C_4_ species, the rate of *g*_sw_ responses to light was slightly, but consistently, greater at *c*_GLA_ compared with *c*_AMB_ and *c*_SUP_. This decreased the difference in Δ*g*_sw_/Δ*t* between C_3_ and C_4_ species. However, a more striking trend, that probably underpinned the subtle difference in relative performance based on photosynthetic type, was that species with higher iWUEs showed greater changes in Δ*g*_sw_/Δ*t* in response to decreasing *c*_a_. At *c*_SUP_, species with high iWUE showed some of the slowest stomatal responses to shade. Because faster stomatal responses are consistent with improved water use efficiency (Lawson and Blatt, 2014), this suggests that transpiration is regulated less tightly at high *c*_a_, supporting the overarching hypothesis that increasing *c*_a_ minimizes the costs associated with hydraulic stress (Polley *et al.*, 1993). It also suggests that characteristics producing high iWUE in the steady state may be costly in low-*c*_a_-like scenarios that increase transpiration. For example, high iWUE is likely to be facilitated by high rates of internal diffusion, which are linked with decreases in cell wall dry matter (Onoda *et al.*, 2017) and might increase vulnerability to changes in leaf water status.

Among-species variation was an important feature of our gas exchange results. This is consistent with previous studies, which have indicated that the degree to which grass stomata protect against decreases in hydraulic conductance varies even among genotypes (Neufeld *et al.*, 1992; Holloway-Phillips and Brodribb, 2011*a*). Among C_3_ species in our study, only that with the highest iWUE, *P. bisulcatum*, showed a clear negative association between *c*_a_ and the stomatal response to shade. At the other extreme, *T. turgidum* showed exceptionally high steady-state *g*_sw_ and slow stomatal responses to shade in all three *c*_a_ treatments, suggesting high transpiration irrespective of leaf water status, a strategy that can maximize CO_2_ uptake at a cost to hydraulic conductance (Holloway-Phillips and Brodribb, 2011*b*). The apparent lack of stomatal regulation in *T. turgidum* compared with other C_3_ species is important to note because iWUE for this species did not decrease at *c*_GLA_, contradicting the otherwise consistent trend towards greater decreases in iWUE among C_3_ compared with C_4_ species.

### Impact of glacial *c*_a_ on LMA and hydraulic characteristics

LMA decreased at *c*_GLA_ among C_3_ but not C_4_ grasses. This finding is consistent with observed differences in *A*, results from a meta-analysis addressing variation in LMA (Poorter *et al.*, 2009), and more recent comparisons using species and *c*_a_ treatments similar to those chosen for our experiment (Pinto *et al.*, 2014). Further evidence is needed, however, before this result can be generalized as a photosynthetic type effect. LMA responses can, for example, be modified by temperature (Pinto *et al.*, 2011). It is also important to note that the C_4_ and two of the wild C_3_ species included in our experiment were drawn from one subfamily of the Poaceae: Panicoideae, a broadly mesic-adapted clade (Taub, 2000; Osborne, 2008; Edwards and Smith, 2010; Visser *et al.*, 2014). We expect leaf functional traits to reflect adaptations to habitat, and some major C_4_ lineages are adapted to drier environments than those favoured by the Panicoideae (Taub, 2000; Edwards and Smith, 2010). In addition, LMA responses to *c*_a_ (Pinto *et al.*, 2016) and leaf size (Liu *et al.*, 2012) differ between the Chloridoideae and Panicoideae grass subfamilies. While further work will be needed to establish whether the patterns we observed are general across grass lineages, our findings are directly relevant to crop and crop-related annual grass species from mesic habitats. Taken together with the gas exchange results, differences in LMA indicate that *c*_GLA_ was linked with greater carbon limitation in C_3_ grasses compared with their C_4_ relatives. This is important because differences in carbon supply affecting plant size and allocation at the whole-plant level have previously been highlighted as central to functional contrasts between C_3_ and C_4_ plants (Long, 1999; Atkinson *et al.*, 2016), and influence the mechanisms by which plants acclimate to hydraulic stress (Maseda and Fernández, 2006).

C_3_ and C_4_ grasses showed similar *K*_leaf_, and *K*_leaf_ decreased at *c*_GLA_. The finding that there was no clear difference in *K*_leaf_ between photosynthetic types is consistent with a previous comparison using the high pressure flow meter technique, applied to predominantly perennial, North American prairie grasses (Ocheltree *et al.*, 2014*b*). Both of these results are surprising because the clearest anatomical differences between C_3_ and C_4_ grass lineages are in the ratio of bundle sheath to mesophyll (Hattersley, 1984; Dengler *et al.*, 1994; Christin *et al.*, 2013; Griffiths *et al.*, 2013; Lundgren *et al.*, 2014). Increases in this ratio should decrease hydraulic resistance external to the xylem (Buckley *et al.*, 2015), supporting the hypothesis that differences in leaf hydraulic properties could affect responses to stress imposed by low *c*_a_ and/or water availability (Osborne and Sack, 2012; Griffiths *et al.*, 2013). It is possible that other aspects of C_4_ leaf anatomy or function counteract positive effects of increased bundle sheath ratios on *K*_leaf_ in C_4_ grasses. It is also important to note that C_3_ and C_4_ grasses often show similar average mesophyll cell sizes at ambient CO_2_ (Lundgren *et al.*, 2014), and the cross-sectional area of vascular relative to chlorenchyma tissues does not necessarily change with photosynthetic type (Dengler *et al.*, 1994).

The evidence we found for decreased *K*_leaf_ at *c*_GLA_ was surprising, because xylem conductivity generally increases with declining *c*_a_ to support increased *g*_sw_ (Rico *et al.*, 2013; Domec *et al.*, 2017). Previous *in situ* measurements of transpiration and leaf water potential in sunflower plants grown at *c*_a_ similar to *c*_GLA_ and *c*_AMB_ showed the expected result: that *K*_leaf_ measured at ambient CO_2_ increased for plants grown at *c*_GLA_, minimizing the impact of increased *g*_sw_ on ΔΨ (Simonin *et al.*, 2015). A decrease in xylem conductivity, linked with smaller conduits in water-stressed tissue that would increase redundancy among conducting elements (Comstock and Sperry, 2000), might contribute to decreases in *K*_leaf_ for leaves grown at *c*_GLA_. However, this is not consistent with the decrease in LMA that we observed and, since transpiration was driven using moderate levels of light, we expect that the primary source of hydraulic resistance was exterior to the xylem (Ocheltree *et al.*, 2014*a*).

The values of *K*_leaf_ were low compared with other recently published estimates for similar species [*S. bicolor*, 19–38 mmol m^−2^ s^−2^ MPa^−1^ (Ocheltree *et al.*, 2014*a*); *O. sativa* cultivars, 7.1–8.7 mmol m^−2^ s^−2^ MPa^−1^ (Xiong *et al.*, 2015)], but are within the range reported in the literature for grasses (~0.44–51 mmol m^−2^ s^−2^ MPa^−1^; Holloway-Phillips and Brodribb, 2011*a*; Ocheltree *et al.*, 2014*a*, *b*; Liu and Osborne, 2015; Xiong *et al.*, 2015) and may be a consequence of moderate PPFD during growth and measurements (Cochard *et al.*, 2007; Ocheltree *et al.*, 2014*a*). Further experimentation and comparison of methods is needed for measurements of *K*_leaf_ in grasses. We need to understand why measurements of *K*_leaf_ produce similar values for C_3_ and C_4_ species; to establish whether *K*_leaf_ responses to *c*_a_ correspond to changes in hydraulic vulnerability; and to determine the anatomical basis of adjustments to *K*_leaf_, especially given evidence for declining LDMC and LMA among C_3_ species at *c*_GLA_. It will also be important to measure *K*_leaf_ at different [CO_2_]; as in the study of *K*_leaf_ responses to *c*_a_ that used sunflower (Simonin *et al.*, 2015), we measured *K*_leaf_ at ambient [CO_2_].

Effects of *c*_a_ on P–V characteristics also provide motivation for further investigation of photosynthetic type×*c*_a_ responses. As leaf size decreased at *c*_GLA_, C_4_ grasses maintained LDMC and C_3_ grasses did not. In parallel, π_TLP_ of C_4_ grasses became more negative at *c*_GLA_, while π_TLP_ of C_3_ grasses did not change. This is an important result because π_TLP_ is a powerful indicator of physiological responses that is expected to integrate smaller changes in, for example, π_0_ and ε (Bartlett *et al.*, 2012). The decrease in LDMC shown by C_3_ leaves grown at *c*_GLA_ is consistent with both lower *A* and LMA, and previous evidence that C_3_ leaves decrease mesophyll cell volume and total non-structural carbohydrates as *c*_a_ declines (Poorter *et al.*, 2009). Maintenance of LDMC and more negative π_TLP_ in C_4_ grasses therefore might be linked with solute accumulation at *c*_GLA_. Presumably, decreases in π_TLP_ of C_4_ leaves at low *c*_a_ would support maintenance of turgor in the presence of larger ΔΨ induced by higher *g*_sw_ (Franks, 2006; Simonin *et al.*, 2015); however, we do not know how leaf-level changes were integrated with adjustments in root and stem properties. The lack of an adjustment in π_TLP_ by C_3_ grasses grown at *c*_GLA_ might be associated with maintenance of leaf water status if root and stem xylem hydraulic conductivity increased or xylem solute concentrations decreased.

### Conclusions

We predicted that gas exchange would show greater absolute responses to *c*_a_ in C_3_ compared with C_4_ grass leaves, especially in terms of the positive relationship between iWUE and *c*_a_. We also predicted that low iWUE at *c*_GLA_ would be linked with changes in leaf hydraulic properties. We found that while the iWUE advantage of some C_4_ grass leaves increased in absolute terms at *c*_SUP_, co-ordination among leaf traits was more strongly affected by *c*_GLA_ than by *c*_SUP_. These experimental results broadly support predicted smaller impacts of *c*_GLA_ on performance of C_4_ grasses (Osborne and Sack, 2012), and suggest that iWUE advantages to C_4_ species will continue to be important in future. A finding with potential importance for crop improvement programmes is that as *c*_a_ increases, pressure on plants to improve iWUE through rapid stomatal responses to shade may be reduced, particularly for species capable of achieving high iWUE. These results highlight the need for continued efforts to establish how hydraulics and photosynthetic performance are co-ordinated, both within leaves and at the scale of whole plants. The mechanistic basis of these responses still needs to be better understood to predict the physiological implications of C_4_ photosynthesis, both under past glacial climates and as they will affect performance in a future high CO_2_ world.
